# Cases of Fractures

**Published:** 1857-01

**Authors:** Frank Hastings Hamilton

**Affiliations:** Buffalo, N. Y.


### BUFFALO MEDICAL JOURNAL

AND

### MONTHLY REVIEW.

**VOL. 12.** JANUARY, 1857. **NO. 8.**

### ORIGINAL COMMUNICATIONS.

ART. I.— *Cases of Fractures.* Reported by FRANK HASTINGS HAMILTON, M. D., Buffalo, N. Y.

(Continued from Page 412.)

### FRACTURES OF THE PATELLA.

CASE I.—*Simple fracture. Perfect bony union.*

Charles Jones, æt. 5, fell on his left knee, Jan. 31, 1848, breaking off a small fragment from the upper and inner margin of the patella. It did not separate from the lower fragment, except that when the knee was bent it was thrown directly forward, presenting to the finger a sharp point.

Dr. Flint and myself in attendance. We directed the lad to keep his leg straight, and to apply cool water dressings.

Six months from this no traces of the accident remained.

CASE II.—*Simple, transverse fracture. Ligamentous union.*

Edward Laffy, æt. 20, fractured the right patella transversely, Oct. 24th,

1851. Dr. Shaw, of Silver Creek, N. Y., dressed the limb with a roller extending from the toes to the knee, and with Sir Astley Cooper's patella apparatus.

He was admitted to the Buffalo Hospital, Nov. 1, 1851, and, with slight modifications, the same dressings were continued until Nov. 26. I then removed the bandage and substituted my own splint, with adhesive plasters.

Dec. 5, I removed the splint and roller from the leg.

The fragments united by ligament; but the ligament was not more than one quarter of an inch in length.

CASE III. — *Simple, transverse fracture. Union by ligament.*

John Delaney, æt. 36, fell, March 7, 1851, about fifteen feet, striking on his head and knee, and breaking the patella transversely.

March 21, he was admitted to the hospital. I immediately applied my own patella splint, securing the foot to the foot-board with a paste gaitre, and supporting the fragments in place with a paste knee cap.

The fragments united by ligament, being separated from each other about one quarter of an inch.

CASE IV. — *Simple fracture. Union by ligament.*

An Irish laborer, fractured the patella transversely, by falling over a dry goods box while running at full speed. Dr. Mixer in attendance.

He was placed upon my splint — a single inclined plane — and the patella retained in place by a strap instead of rollers.

At the request of Dr. Mixer, I saw him occasionally during the treatment.

Four months after the injury, the functions of the limb were completely restored, union of the fragments having been effected with a fibro-ligamentous tissue half an inch in length.

CASE V. — *Simple fracture. Union by ligament.*

Theophilus Alles, æt. 56, fell on the sidewalk, Aug. 27, 1854, breaking the patella of the left leg. Fracture transverse and simple.

The surgeon who was employed, applied only bandages to the leg and knee. No splint was used.

Sept. 10, 1854, two weeks after the fracture occurred, he was received into the Buffalo Hospital, and the same treatment was continued.

Oct. 1, 1854, when I took charge of the wards, I found the fragments united by ligament, but separated half an inch. On the 6th, I removed all dressings.

Case VI. — *Simple, transverse fracture. Union by ligament.*

James McCuen, æt. 33, fractured the left patella transversely. Dr. Mixer and myself in attendance. The splint and the other dressings were continued three weeks and three days.

After two years I find the patella united by a ligament of half an inch in length. The limb is not quite as strong as the other.

Case VII. — *Simple, transverse fracture, from muscular action. Union by ligament.*

Oct. 21, 1846, a sailor was walking across the floor when his heel caught in a knot hole and threw him backward, breaking the patella transversely. The fracture was the result of muscular action alone.

I took charge of the case, at the request of Dr. Flint, on the third or fourth day. We applied my patella splint in the usual manner.

In four weeks ligamentous union was accomplished, with a separation of the fragments to the distance of half an inch.

Case VIII. — *Simple, transverse fracture. Union by ligament. Abundant callus. Partial rupture of ligament.*

Wheeler, æt. 40, fractured the patella transversely. Fracture treated by Dr. Williams.

Nine weeks after the fracture occurred, I examined the knee. The patella was surrounded with bony callus, so that it was considerably wider than the other. The fragments seemed to be united by a short ligament, except on the inner side, where there was a separation or rupture of the ligament, to the extent of one quarter of an inch. The patient explains this by saying that the splint was removed at the end of four weeks, and that after a week more he began to walk, but that he almost immediately felt it tear or give way on the inner side.

Cases IX and X.—*Simple fracture of both patellas. Union by ligament.*

John Dundas, æt. 22, fell, Oct. 22, 1852, in the night, while asleep, from a window in the third story of a dwelling-house, striking with his knees upon the stone sidewalk.

He was seen by Dr. Pratt, and Gibson's long thigh splints were applied. On the tenth day I took charge of him at the Buffalo Hospital, and substituted my simple inclined plane for the Gibson splint. I covered each limb from the toes upward, as far as the knees, with a paste bandage, and then, having properly cushioned the limbs, I secured the fragments in place with adhesive plaster.

Nov. 28, 1852, thirty-seven days after the fractures occurred, all dressings were removed. Both patellas had united by ligaments of about one quarter of an inch in length.

Case XI.—*Simple, transverse fracture. Occurring twice at the same point. Union by ligament.*

Carl Abend, æt. 30, fractured his left patella, Dec. 4, 1850, and again Feb. 3, 1851.

He was admitted to the Buffalo Hospital of the Sisters of Charity, Feb. 6, 1851. The fracture was transverse, and the fragments were separated one inch. The whole limb was swollen, and as the splint had now been on the limb a great portion of the time since the first fracture, I had it removed altogether, and directed the limb to be bathed twice daily, with cold water. This treatment was continued ten days. I then applied my own splint.

March 21, six weeks from the date of the last fracture, I removed all dressings and found the fragments united by a ligament of one quarter of an inch in length. (See cast in my cabinet.)

Case XII.—*Simple fracture. No union of the fragments.*

John Sharkie, æt. 24, a soldier in the British service, while serving in the East Indies, was struck on the right knee while he was in a sitting posture, with his leg bent under him.

He was immediately placed under the charge of the surgeon of the 89th Regt. of Infantry. During the first eleven days no splints or bandages were applied, on account of the severe inflammation and swelling. A compress was then placed over both fragments, and they were bound together by rollers, &c. The whole limb was suspended on an inclined plane, the foot being made fast to a foot board. This treatment was continued four months. When the bandages were removed the limb was badly swollen. Immediately on removing the bandages the upper fragments drew up toward the body. It was eighteen months before he could walk, even with the aid of a cane.

March 27th, 1855, twenty-nine years after the injury was received, he was an inmate of the Buffalo Hospital, and I was permitted to examine his knee carefully.

The lower fragment is not displaced, but when the leg is straight upon the thigh, the upper fragment lies two and a-half inches from the lower, and when it is flexed upon the thigh, the upper fragment is removed five inches from the lower. There is no ligament or other bond of union, so far as I can discover. He walks with very little or no halt, but he cannot walk fast.

Case XIII.— *Comminuted fracture, complicated with fracture of the Femur. Result imperfect.*

Dec. 27, 1853, Wm. Palmerston, of Black Rock, Erie Co., N. Y., about 25 years old, fell, and broke both his left thigh and patella, (See Case LXX of "fractures of femur.") The patella was broken, transversely, near its middle. There was also a longitudinal fracture near the inner margin.

Drs. Lewis and Dayton were in attendance. I was requested by these gentlemen to see the case on the fifth day. The femur being broken, also, our treatment was, in consequence, somewhat modified. A single straight splint was employed and adhesive straps were laid over the patella.

On the fifty eighth day the splint was not yet removed from his knee, but the fragments were united.

June 3, 1854, five months after the fracture occurred, I found the fragments separated half an inch, but united by what felt like bone. There was a small loose fragment on one side, and which occasionally produced pain and swelling at that point. He had but little motion in the knee-joint, yet he was able to walk and to pursue his trade as a carpenter, without much inconvenience.

### TIBIA.

#### UPPER THIRD.

CASE I.—*Simple, transverse fracture. Result perfect.*

Mrs. W., of Buffalo, æt. 58, fell, Oct. 19, 1848, striking on her right knee, breaking the tibia just below the tuberosity. Fracture transverse. The fall was the result of a misstep on level ground, and was attended with only slight bruising of the soft parts. She says, that on attempting to rise she discovered what had happened, the bone projecting very distinctly, and she pushed and pulled it into place with her own hands.

Dr. Barnes, who was the family physician, requested me to see it on the same day. Mrs. W. was large, with a leuco-phlegmatic temperament. The limb was already swollen and œdematous. The fragments were in place, but motion and crepitus were distinct.

We dressed the limb by laying it on a pillow, and supporting it with two lateral splints placed outside of the pillow, and confined the limb to the pillow with tapes. About one week from this, we laid the limb over a double inclined plane. The fragments united promptly and without the slightest amount of displacement or deformity, or ensheathing callus. In six weeks all dressings were discontinued.

The neck of the femur of the same limb, was broken in 1841. (See Case XVI. of "fractures of femur.")

CASE II.—*Simple, transverse fracture. Result perfect.*

Peter Hamil, of Buffalo, æt. 29, was admitted to the hospital, Aug. 31st, 1849, with an injury to his left leg, which had occurred two days before. A young surgeon in the city had examined the limb, and thought the femur was broken just above the joint. He had applied a roller from the toes to the thigh, and to the thigh were applied very nicely adjusted lateral splints. These dressings were on the limb at the time of his admission, and were not removed until the next day. I could not then discover any fracture or displacement, and the dressings were discontinued, the limb being merely laid upon pillows. After about eight days, however, when the swelling of the foot, consequent upon the bandaging, had subsided, I reäpplied side splints, believing it possible that I might have overlooked a transverse fracture of the lower end of the femur. On the 26th of Sept., I discontinued them altogether.

Oct. 4, when examining the limb, I found a slipping sensation, like that produced in a false joint, through the upper end of the tibia, and I now easily understood what had been mistaken for a fracture of the femur. It was a transverse fracture through the upper end of the tibia, and without displacement. Union was delayed, perhaps owing to its not having been at all supported by splints. The knee-joint was quite stiff. No splints were afterward applied, and on the 25th of Nov., three months after admission, he was dismissed, the motion between the fragments having ceased, but the knee still remaining quite stiff.

CASE III.—*Simple fracture. Result perfect.*

James Ellis, æt. 42, broke the tibia about two or three inches below its articulating surface. It was treated by a surgeon at Port Sarnia, C. W.

Ten weeks after the accident he called upon me, and I found the bone united, but some bent at the seat of fracture. In all other respects it was perfect.

CASE IV.—*Simple fracture. Result perfect.*

John G. Zimmerman, a citizen of Saxony, in Germany. When eight years old he fractured the left tibia about three inches below the knee. The fracture was examined by two surgeons, both of whom pronounced it a fracture. The fragments were moveable. They applied lateral splints and bandages.

March 31, 1854, while Zimmerman was an inmate of the Buffalo Hospital, and thirty-seven years after the accident, I examined the limb and found no traces of a fracture.

CASE V.—*Simple fracture, with displacement of head of fibula. Result imperfect.*

Robert Purcell, of Attica, N. Y., æt. 55, broke the tibia about three inches from the knee-joint; at the same time displacing the upper end of the fibula upward.

Dr. Curtis, of Attica, N. Y., was employed.

Two years after the accident I found the limb shortened one-quarter of an inch; the tibia slightly bent at the seat of fracture; the head of the fibula remaining displaced, and the limb painful when he walked much.

The same leg was broken in its middle two years before. (See Case XIV of "fractures of tibia.")

MIDDLE THIRD.

Case VI. — *Simple, transverse fracture. Result perfect.*

July 6, 1849, Mrs. Lathan, of Buffalo, æt. 50, broke the left tibia near its middle. Fracture transverse, and never displaced. I dressed the limb at first temporarily, and on the fifth day I applied a starch bandage. At the end of two months union was complete and without displacement.

Case VII. — *Simple fracture. Result perfect.*

R. Jackson, of Buffalo, æt. 46, broke the tibia near its middle. Does not know the name of the surgeon who dressed the fracture.

Ten months after, the limb was of the same length with the other, but there was a slight forward bend at the seat of fracture, and the limb still pained him occasionally.

Case VIII. — *Simple, transverse fracture. Result perfect.*

George Peterson, of Buffalo, æt. 11, fractured his right tibia near its middle, June 25, 1850, by falling from a building, twenty feet. Fracture transverse, and fragments not displaced. Very large thrombus. I laid the limb simply on a pillow until the sixth day, when I applied a paste roller. On the twenty-fifth day after the fracture, I dismissed him with a perfect cure.

Six years after, the limb was perfect.

Case IX. — *Simple fracture. Result perfect.*

George Eggleston, æt. 17, fractured the right tibia near its middle. The fracture was treated by Dr. Blair, of Rome, N. Y.

Eleven years after, when examined by myself, the limb was sound and perfect.

Case X. — *Simple fracture. Result perfect.*

Michael Hollis, æt. 30, fractured the tibia of his left leg near its middle. Dr. Crandall, of Palmyra, treated the fracture.

Four years after, when examined by myself, the limb was perfect.

Case XI.—*Simple fracture. Result perfect.*

Wilhelm Hansikom, æt. 24, broke the tibia near the lower end of the middle third. It was dressed by Dr. Walter, of New Brandenburg, Germany. A large number of narrow splints were placed around the limb.

Jan. 8, 1856, thirty years after the accident, and while he was an inmate of the Buffalo Hospital, I examined the leg. It was in every respect perfect.

Case XII.—*Simple fracture. Result perfect.*

Harrison Webster, æt. 17, broke the tibia near its middle. Dr. Needham, of Onondaga, N. Y., treated the fracture.

Four years after the accident, the limb was perfect.

Case XIII.—*Simple fracture. Result perfect.*

Jeremiah Torrey, æt. 11, broke the right tibia through its middle. Surgeon Day, of England, treated the fracture.

After sixteen years I found the limb sound and without deformity.

Case XIV.—*Compound fracture. Result perfect.*

Robert Purcell, æt. 53, fractured the tibia through its lower third. Dr. Curtis, of Attica, N. Y., was employed.

Four years after the fracture occurred I found no traces of the fracture.

Case XV.—*Compound fracture. Result imperfect.*

Hugh Boyle, of Toronto, C. W., æt. 26. Fracture of the right tibia, near its middle. Dr. Witmer, of Toronto, took charge of the limb.

Nine years after, he became an inmate of the Buffalo Hospital, on account of an ulcer which had existed over the seat of fracture ever since the accident occurred.

Case XVI.—*Compound fracture. Result imperfect.*

John Mahan, æt. 39. Admitted to the Buffalo Hospital, Feb. 16, 1853, with a compound fracture of the right tibia, near the middle of the leg. The bone was broken by the kick of a Dutchman. I found the limb much swollen, and very painful, and I laid it carefully over a double inclined plane, and directed cold water irrigations; I also directed morphine in full doses. The inflammation, for several days, threatened the complete loss of his limb. On the tenth day the lower end of the upper fragment was projecting in front of the lower fragment, and I covered the angle of the splint and made moderate pressure upon the upper fragment. On the twentieth day the fragments were bent backward, and I placed a compress behind. On the thirty-seventh day we took the limb from the inclined plane, and trusted alone to side splints. On the forty-fifth day we removed all dressings. Bone not united. Laid the limb simply on a pillow. After six days I applied a moveable gutta percha splint. Directed the limb to be bathed and rubbed every day, and allowed patient to sit up. Fifty-ninth day, he began to walk on crutches. Motion between fragments still continued. On the ninety-third day I found a firm union, without ensheathing callus. The upper fragment is in front of the lower.

Case XVII.— *Compound, comminuted fracture. Result imperfect.*

Simon Cone, of Buffalo, æt. 28, broke his —— tibia through its middle third. Dr. Hauenstein was employed.

Seven months after the accident occurred, I removed a small fragment which had never united to the shaft. The wound had closed over, and for a long time this fragment could be felt moving under the skin, but not causing inflammation or suppuration, yet the skin over it was tender, and he wished it removed.

The leg was shortened one-quarter of an inch. I did not, however, detect any displacement of the fibula, yet it must have existed.

### MALLEOLUS INTERNUS.

#### LOWER THIRD.

Case XVIII.— *Compound fracture, complicated with fracture of astragalus. Result imperfect.*

Pat. Murphy, æt. 28, admitted to the Hospital of the Sisters of Charity, Oct. 22, 1848.

"Three weeks since, his right foot was traversed by the wheel of an empty car, and badly crushed. Until received here, he had been under the care of Dr. Erastus Wallis, of this city; and the swelling, at first very great, had been considerably reduced, and the wounds partially healed. Crepitus was distinct upon slight motion of foot. A gutta percha splint was applied to the leg and foot, with also simple dressings to the wounds, and the whole limb was placed in a Bowen's splint.

"From this date until the 31st, the discharge of matter was profuse, and often highly offensive. He suffered much from pain and general restlessness. Night sweats and occasional chills, also, with emaciation, indicated an approach to hectic. The treatment was chiefly tonic and stimulating. Under this plan, with careful dressing and management of the leg, he gradually passed the crisis, and from about the 31st of October, more than four weeks after the accident, he slowly improved. Several fragments of bone were discharged, but when dismissed, Dec. 12th, he had tolerably free use of his ankle-joint, the wounds had healed, and he had a very useful, but not perfect limb.

"We never absolutely condemned this limb to amputation, yet, at one time, we warned him of the probability that it would soon be necessary to make this sacrifice for the safety of his life."

### ABOVE MALLEOLUS.

#### LOWER THIRD.

CASE XIX.—*Simple fracture. Result perfect.*

James McDonald, of Buffalo, æt. 33, fractured the right tibia about two inches above the ankle-joint. It was treated by Dr. Austin Flint, of Buffalo.

Three years afterward I could find no traces of the accident.

CASE XX.—*Compound fracture. (A gunshot wound.) Result imperfect.*

John Thurstone, æt. 17, was shot in the left leg, breaking the tibia about two or three inches above the ankle-joint. Dr. Amasa Trowbridge, of Watertown, N. Y., was employed.

Six years after I found the bone united with some deformity, but not shortened.

(To be Continued.)

